# The Cognitive Approach to Bioethical Issues in Perinatal Care in Greece

**DOI:** 10.7759/cureus.22760

**Published:** 2022-03-01

**Authors:** Anna Glynou, Elena Frysira, Kalliopi Christakakou-Fotiadi, Makarios Eleftheriadis, Angeliki Sarella, Iosifina Stergiotou, Maria Koukaki, Eirini Chasalevri, Dionysios Galatis, Nicolaos Salakos

**Affiliations:** 1 Fourth Department of Nursing/Midwifery, Maternity Hospital Elena Venizelou, Athens, GRC; 2 Laboratory of Medical Genetics, National and Kapodistrian University of Athens, Athens, GRC; 3 Department of Law, National and Kapodistrian University of Athens, Athens, GRC; 4 Second Department of Obstetrics and Gynecology, Aretaieion Hospital, National and Kapodistrian University of Athens, Athens, GRC; 5 Department of Midwifery, Faculty of Health and Caring Sciences, University of West Attica, Athens, GRC; 6 Department of Obstetrics and Gynecology, Fetal Medicine Center, Corfu, GRC; 7 Department of Obstetrics and Gynecology, Maternity Hospital Leto, Athens, GRC; 8 Department of Obstetrics and Gynecology, National and Kapodistrian University of Athens School of Medicine, Athens, GRC

**Keywords:** bioethics, health professionals education, informed consent, prenatal counselling, bioethical dilemmas

## Abstract

Background and aim: Current practice in prenatal diagnosis becomes challenging with new bioethics issues emerging constantly during daily clinical routine. Although fetal interventions are driven by a motivation to improve the health of the fetus, progress in fetal therapies raises issues of maternal autonomy. The objective of this article is to assess bioethics in prenatal diagnosis in Greece as well as bioethics education.

Methods: The study was conducted between October 2018 and December 2019. Two hundred and twenty eligible responders were involved in fetal and perinatal medicine in Greece. The questionnaire was developed as a Likert scale. Part 1 covered the participants' general opinion about bioethics. Part 2 covered ethical dilemmas likely to arise when routine screening presents a complicated result.

Results: In the study, 92.3% of the participants considered that the branch of bioethics is necessary in medical practice. Regarding challenging bioethics issues, only 86% of the participants consider that the miscarriage risk should be discussed after an invasive procedure. Furthermore, it is not clear for responders whether informed consent is a medical or legal obligation (43% vs 33%) and whether information should be provided orally or written (49% vs 46%). Finally, 32% of healthcare practitioners declare that they are not fully aware of the law concerning the rights of the fetus.

Conclusions: Although healthcare professionals acknowledge the distinct role of bioethics, mismanagement of ethical dilemmas reveals that under-graduate teaching of this discipline is not addressed effectively. Identifying the parameters that would improve the learning process would make a significant contribution in the routine clinical practice.

## Introduction

Recent knowledge and new technological advances in the field of prenatal diagnosis during the last two decades have provided important information about establishing good health for both pregnant women and fetuses [1]. Bioethics, on the other hand, is a discipline in the intersection of many scientific fields, such as Biology, Medicine, Genetics, Biotechnology, and Biomedicine and focuses on ethical issues in medical profession and health care [2]. Furthermore, other disciplines are involved, such as Law and Theology [3].

Current practice becomes challenging with new bioethics issues emerging constantly during daily clinical routine. Prenatal diagnosis for fetal aneuploidies relies on an initial risk assessment screening for trisomy 21, trisomy 18, and trisomy 13 in the first trimester of pregnancy. Screening aims, however, not only at diagnosing genetic abnormalities but also diseases of the fetus throughout pregnancy. The routine offer of medical tests and invasive procedures is recently marked with the advent of fetal therapy destined to improve fetal and neonatal health by intervening in utero to correct or treat prenatally anatomical abnormalities diagnosed by ultrasound [4]. Although fetal interventions are driven by a motivation to improve the health of the fetus and the newborn in the long run, progress in fetal therapies raises issues of maternal autonomy as well as decision making [4].

It is clear that health professionals’ main role in antenatal care is to contribute to the well-being of fetuses and their mothers but also to inform and encourage prospective parents, respecting their autonomy and informed choice [5,6]. Informed consent is the most common practical form of expression of individual autonomy [7]. It is based on sufficient and accurate information provided to pregnant women, with the aim of consciously making decisions [8]. Women considered to be at high risk are routinely offered invasive procedures. At the same time, doctors should promote reproductive autonomy supporting pregnant women’s right to refuse any procedure while maintaining privacy and confidentiality [9]. Health professionals in the field of Obstetrics should take into consideration the influence and the strong emotional impact they have on pregnant women during the particularly sensitive period of pregnancy [8] and they should develop skills to provide the best quality of care.

To date there are not many studies addressing bioethics in Greece and there are no data on bioethics education in universities throughout the country. The objective of this article is to assess how healthcare providers in the field of Obstetrics (obstetricians/gynecologists, geneticists, and midwives) deal with bioethical issues and the current state of bioethics education in the medical and midwifery programs across Greece.

## Materials and methods

This cross-sectional study was conducted between October 2018 and December 2019 in Prenatal Control departments of Greece. A questionnaire was developed by a multidisciplinary group of clinicians. The questionnaire was completed by 220 health professionals (obstetricians/gynecologists, geneticists, and midwives). Before completing the questionnaire, health professionals were given both written information by a study information sheet as well as oral information about the content and purpose of the research. The 220 participants were divided into groups: obstetricians-gynecologists, geneticists, and midwives, working either in the private or public sector, to assess any different views. All eligible responders were involved in fetal and perinatal medicine in Greece. The questionnaire was developed as a Likert scale, which is widely used to measure attitudes and opinions with a greater degree of nuance than a simple 'yes/no' question. It consisted of two sections of both six questions. Part 1 covered the participants' general opinion about bioethics indicating their agreement, disagreement, or uncertainty (ticking ‘not at all likely’, ‘not so likely’, ‘somewhat likely’, ‘very likely’, ‘extremely likely’) for six descriptive statements. Part 2 formulated again as a Likert scale covered perceptions of the ethical concerns likely to arise when routine screening presents a complicated result. The survey was approved by the University of Athens Research Ethics Committee (National Health System Health Administration of Attica Region General Children΄s Hospital "AGIA SOFIA" / Scientific Council : 14904/14-06-18).

Statistical analysis

Descriptive statistics are presented, i.e. responses’ absolute number and the percentage for each multiple choice question were calculated on the total of participants’ answers. The analyses were carried out using the statistical package SPSS version 22 (SPSS Inc., Chicago, IL, USA).

## Results

Demographic and clinical characteristics

The sample consisted of 84 men and 136 women. More specifically, 40% of the sample were obstetricians-gynecologists, 3.2% medical geneticists, 8.6% doctors of other related specialties, and 48.2% were midwives. 61.8% of the participants were women and 31.4% were 39-48 years old. 42.7% were university graduates and 33.6% were postgraduates. Almost all participants (89.5%) had an active employment and in fact 58.1% worked in the private sector and 30.4% worked in a public sector (Table 1).

**Table 1:** Demographic and clinical characteristics of the sample (N = 220)

		N	%
Sex			
	Men	84	38.2
	Women	136	61.8
Age			
	18-28	33	15.0
	29-38	54	24.5
	39-48	69	31.4
	49-58	43	19.5
	59 and older	21	9.5
Educational level			
	Secondary education	2	0.9
	University - Technological education	94	42.7
	Postgraduate education	74	33.6
	Holder of a doctorate or post-doctoral degree	50	22.7
Specialty			
	Obstetrician/gynecologist	88	40.0
	Medical geneticist	7	3.2
	Doctor of other specialty of a related field	19	8.6
	Midwifery staff	106	48.2
Do you work?			
	No	23	10.5
	Yes	197	89.5
If yes, please select one of the following options:			
	Public hospital	55	27.9
	Public sector	12	6.1
	Private clinic	41	20.8
	Private sector	87	44.2
	Other	2	1.0

Questions related to bioethics issues addressed in this study and related answers of the participants are included in Table 2.

**Table 2:** Participants' responses concerning bioethics issues

		N	%
Do you think you know the meaning of the term 'bioethics' and the parameters it entails?			
	Not at all well	3	1.4
	Not so well	18	8.2
	Moderate somewhat well	67	30.5
	Very well	85	38.6
	Extremely likely	47	21.4
Do you think that bioethics is necessary in medical practice?			
	Not at all likely	1	0.5
	Not so likely	2	0.9
	Somewhat likely	14	6.4
	Very likely	71	32.3
	Very much extremely likely	132	60.0
Do you think that your religious beliefs shape your bioethical decisions while attending a pregnancy?			
	Not at all likely	75	34.1
	Not so likely	37	16.8
	Moderate somewhat likely	46	20.9
	Very likely	42	19.1
	Extremely likely	20	9.1
Do you think that the Greek legislative framework can determine your bioethical decisions?			
	Not at all likely	18	8.2
	Not so likely	32	14.5
	Moderate somewhat likely	74	33.6
	Very likely	75	34.1
	Extremely likely	21	9.5
Your source of information on bioethics and dilemmas arising out of your duties derives from:			
	Basic education at undergraduate studies	42	19.1
	Books/journals/articles	62	28.2
	Media	3	1.4
	Conferences, seminars, vocational training courses	76	34.5
	Online information/internet	24	10.9
	Various other sources	13	5.9
If you were asked to address a bioethical dilemma related to pregnancy, which of the following sources would you initially address?			
	The Medical Association - Midwives Association	17	7.7
	The Scientific Board of the Hospital	28	12.7
	Lawyer	12	5.5
	Colleagues/Advisory Group	111	50.5
	Bibliographic sources	52	23.6

Sixty percent of the participants considered that they know very well the meaning of the term 'bioethics' and the parameters it includes and 92.3% considered that the branch of bioethics is necessary in medical practice. Twenty-eight percent of participants felt that their religious beliefs determine their decisions on bioethical issues when monitoring a pregnancy. In addition, 43.6% of the participants considered that the Greek legal framework could determine their decisions on bioethics. The main source of information on bioethics and the dilemmas that arise during the performance of the participants' duties were conferences, seminars, vocational training courses at a rate of 34.5%, followed by books, scientific journals, and articles at a rate of almost 30%.

**Figure 1:**
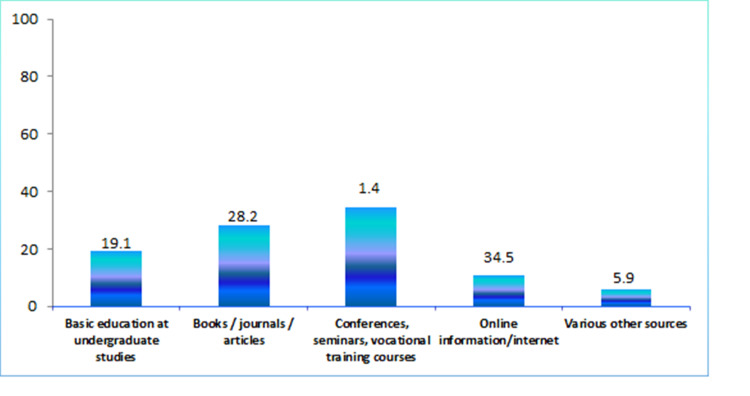
Sources of information on bioethics and the dilemmas that arise during the performance of duties

In cases where responders were asked to deal with a bioethical dilemma related to pregnancy, most of them (50.5%) turned to a colleague and 23.6% to literature. Figure 1 gives the sources of information regarding bioethics and the dilemmas that arise during the performance of the participants' duties.

Table 3 presents the participants’ beliefs on further prenatal diagnosis issues. Fifty percent of them think that termination of pregnancy could be a benefit of prenatal diagnosis, while 86% consider that doctors should discuss with the patients the risk of miscarriage after an invasive procedure. On the other hand, it seems that it is not clear for responders whether informed consent is a medical or legal obligation (43% vs 33%) and whether information should be provided orally or written (49% vs 46%). Finally, 32% of healthcare practitioners declare that they are not fully aware of the law concerning the rights of the fetus.

**Table 3:** Participants' beliefs concerning prenatal diagnosis issues

		N	%
Do you think that the prenatal diagnosis is an important parameter in the care of the pregnant woman?			
	Not at all well	1	0.5
	Not so well	2	0.9
	Moderate Somewhat well	6	2.7
	Very well	29	13.2
	Extremely likely	182	82.7
Do you think that a possible benefit of the prenatal diagnosis is:			
	The possibility of legal termination of pregnancy	14	6.4
	Terminating the pregnancy when the woman wishes so	14	6.4
	Termination of pregnancy where there is a likelihood of serious disability or illness for the fetus	110	50.0
	Prenatal diagnosis refers to the prevention and not only the possibility of termination of pregnancy	82	37.3
Do you believe that pregnant women should be thoroughly informed about the possibility of miscarriage when performing invasive prenatal testing, such as chorionic villus sampling and amniocentesis?			
	Not at all likely	0	0.0
	Not so likely	0	0.0
	Somewhat likely	1	0.5
	Very likely	25	11.4
	Extremely likely	194	88.2
In your opinion, the informed consent, as applicable in Greece, is:			
	A medical obligation	96	43.6
	A legal obligation	74	33.6
	A moral obligation	49	22.3
	Not necessary	1	0.5
Do you agree with the legislation on the concept of 'fetus as a patient' and 'right to life'?			
	Not at all likely	6	2.7
	Not so likely	11	5.0
	Somewhat likely	43	19.5
	Very likely	48	21.8
	Extremely likely	42	19.1
	I do not think I am fully aware of this issue	70	31.8
What is considered to be the most appropriate way to report adverse effects in the prenatal screening?			
	Verbally with complete and accurate information	9	4.1
	Verbally with full details addressing the patient's emotional response, with discussion and future planning	108	49.1
	In writing, in full detail by addressing the patient's emotional response, with discussion and future planning	103	46.8

## Discussion

A global trend in medicine acknowledges the introduction of bioethical questions in its routine practice. Prenatal screening and diagnosis have experienced an unprecedented progress through the advances in biomedical technology, genetics, and therapeutic procedures in utero. Any intervention on the fetus can certainly affect the well-being of both fetus and pregnant woman and determine their prospective future.

It is crucial to have in mind that prenatal counseling has four main purposes: 1) to inform parents about the genetic constitution of the fetus and guide them appropriately for the birth of a newborn with a disability, 2) to present the possibilities of immediate or after-birth intervention and/or treatment, 3) to list all possible options and risks, and 4) to assess the option of termination of pregnancy depending on the severity of the fetal condition. The healthcare professionals involved in prenatal care should be aware of their need for continuous scientific training and ensure the acquisition of specific emotional skills to deal with bioethical issues while respecting maternal autonomy, and the informed consent process [4].

In the present survey, 92.3% of the respondents considered that the field of bioethics is necessary in medical practice while only 60% of the participants considered that they know very well the meaning of the term 'bioethics' and the parameters it includes. They also believe that the personal values, religious beliefs, and moral values of the parents should be taken into account and that the health professionals should focus on the specific character of each family and the individualized treatment of every suffering fetus. Although they acknowledge that experts should not rely on their personal views, they admit that they are not fully informed of the existing law and that they rather provide oral rather than written information. Furthermore, it is interesting to note that 50% of the participants prefer anticipating the opinion of an experienced colleague or advisory body, while undergraduate education is not obviously considered important enough to offer didactic tools to healthcare professionals. According to our research, only 19% of the participants refer to some undergraduate training in bioethics, while 34% acquired knowledge by a postgraduate modality. Additionally, healthcare professionals have a vague consideration of the prenatal diagnosis purpose. Our research showed that 28% of the participants considered that their religious beliefs determine their decisions on bioethical issues when monitoring pregnancy. What is more, there is no consistency on the proper way to communicate bad or uncertain news to parents.

Coordination in care delivery, good cooperation, and communication between health professionals are very important and can minimize conflicting views in their decisions. Only multidisciplinary teams deal successfully with complex bioethical issues. Prenatal screening centers should employ health professionals specializing in various fields, such as fetal medicine experts, geneticists, midwives, pediatric cardiologists and surgeons, lawyers, and members of institutional ethics committees [4]. Complex healthcare issues require specialized experience and training [10]. This necessity is not always met with due attention on the part of the Greek academic system. Uncertainty in bioethics education seems to be a global issue though. In Western countries educational methods still do not advance in the same way. Despite controversy, however, it is clear that ‘ethics can indeed be taught'; it deals with rendering specific judgment and action despite complexity and uncertainty [6]. It is interesting to note that GMC in its 2009 version of Tomorrow’s Doctor refers to that requirement of the medical school graduate to be able to behave according to ethical and legal principles and to know about and keep to the GMC’s ethical guidance and standards.

**Figure 2:**
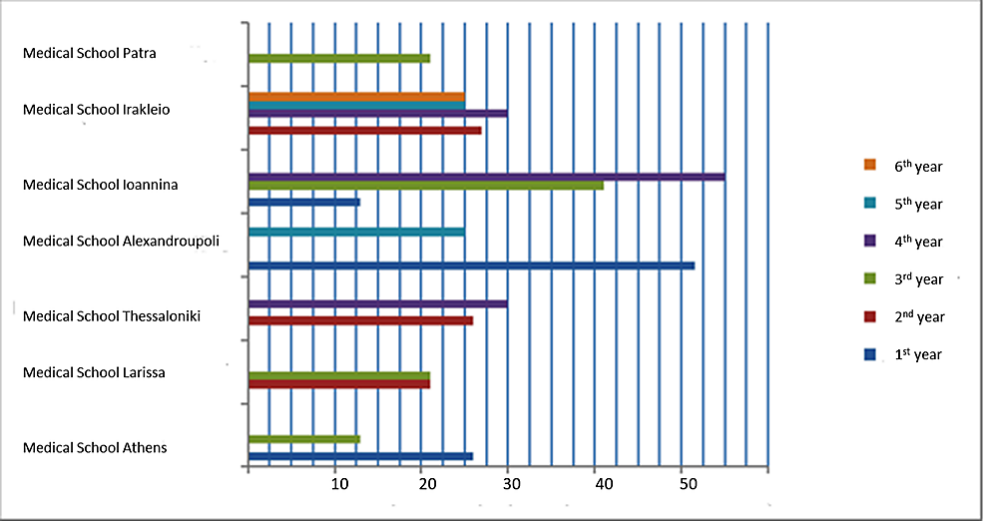
Demonstrates bioethics education in medical schools in Greek universities

**Figure 3:**
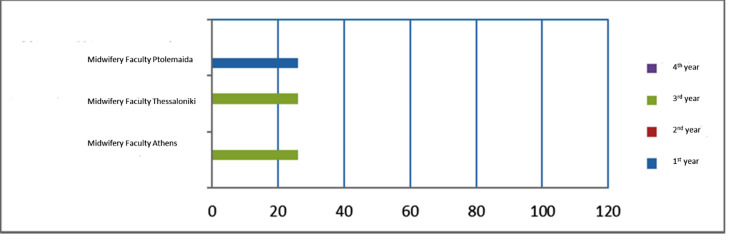
Demonstrates bioethics education in midwifery faculties in Greek universities

Unlike many countries such as the UK and USA which have experienced years of strict and systematic didactic methodologies in bioethics, its teaching in Greece has only been recently introduced in medical curricula. Furthermore, no academic guidelines for bioethics education have been established [11-13]. Τhis fact explains why only 29% of our participants refer to their undergraduate training. Most of them never had the chance to acquire a systematic approach of bioethics and, therefore, needed to attend an additional optional training activity or rely on the expert opinion of a colleague or an advisory body. At the educational level, a heterogeneity was noted both on the type of course and the teaching mode. It is obvious that the didactic tools are left on the discretion of the academics. Specifically, we investigated the curricula of medical schools and midwifery faculties in Greece to assess their current agenda on bioethics (Figures 2, 3). In most cases, bioethics is combined with training on the legal background for each profession. Interestingly, bioethics in one institution is combined with history of medicine and epistemology teaching, while in another two medical schools bioethics teaching is only poorly represented in its curriculum; bioethics is taught in one semester and the duration of the didactic hours is an average 25 hours. Additionally, there is no agreement on the timing of teaching throughout the curricula. Mostly details are not provided on the teaching method, the didactic approach involved in this particular discipline, and the type of assessment. It is not also mentioned if bioethics learning is delivered by an interdisciplinary team. A plurality of approaches should have been encouraged. On the other side, a similar situation is held in the professional training of midwives with bioethics teaching being optional in one faculty and mandatory in the other two institutions. Likewise, no further information is provided on the actual educational level and activities. It is interesting to note that two postgraduate courses are available in Greece as part of the continuing educational process. Bioethical competence is delivered both by clinicians and lawyers so that a medical legal perspective is provided to those postgraduate students who wish to acquire a diploma on bioethics. It would be important to mention that the availability of scholars with an ethics background is always limited [14]. However, medical schools should set high academic standards and involve several disciplines in the bioethics teaching. It is of great importance to involve academics from distinct educational backgrounds, given the multidisciplinary nature of bioethics [15].

Learning and teaching of medical ethics are incomparably better than they were 20 years ago. However, much still needs to be done to achieve a level of competence [16]. In the field of prenatal diagnosis in Greece, as our study demonstrates, continuing education should be encouraged due to the limited expertise of healthcare professionals to deal with complex bioethical issues. To overcome the limits of the undergraduate curricula and their overloaded schedule, all academics should try to stimulate a constant teaching strategy with the aim to improve students’ analytical skills [17]. Professional societies and advisory bodies of this field should promote educational methods that enhance support, and care to families in need of prenatal diagnosis, while teaching a sensitive counselling approach respecting patients’ autonomy and privacy. To the best of our knowledge, this is one of the first national surveys assessing bioethics in prenatal diagnosis in Greece [18]. Furthermore, findings should give national academics an insight into the current state of bioethics education in Greek universities. However, a few limitations need to be considered. Only healthcare professionals involved in prenatal care participated in the study. Furthermore, the sample size of the participants is limited, and their opinion possibly is not representative of the experience and needs of healthcare professionals of different disciplines throughout the country. Therefore, conclusions cannot be generalized and further studies need to be designed to assess the current perception of bioethics and the teaching approach of this discipline in Greece.

## Conclusions

In conclusion, our study suggests that healthcare professionals acknowledge the distinct role of bioethics in prenatal diagnosis. However, mismanagement of ethical dilemmas reveals that under- and post-graduate teaching of this discipline is not addressed effectively. Identifying the parameters that would improve the learning process would make a significant contribution in the routine clinical practice by consolidating bioethical principles and appropriate counseling techniques.
